# The complete chloroplast genome of *Barringtonia asiatica* (L.) Kurz (Lecythidaceae)

**DOI:** 10.1080/23802359.2025.2457456

**Published:** 2025-01-30

**Authors:** Emmanuel Bonifacio S. Timog, Renerio P. Gentallan, Kristine Joyce O. Quiñones, Michael Cedric B. Bartolome, Daryl B. Ceribo

**Affiliations:** a Crop Breeding and Genetic Resources Division, Institute of Crop Science, College of Agriculture and Food Science, University of the Philippines Los Baños, Laguna, Philippines; b Department of Forest Biological Sciences, College of Forestry and Natural Resources, University of the Philippines Los Baños, Laguna, Philippines; c Department of Science and Technology, Science Education Institute, Manila, Philippines

**Keywords:** Botong, medicinal tree, phylogeny, Planchonioideae, plastome

## Abstract

Genomic resources for *Barringtonia asiatica* (L.) Kurz, a pantropical beach forest species native to the Philippines, have not been published to date, despite its medicinal importance and potential for biomedical applications. In this study, we assembled and characterized the first complete chloroplast genome of *B. asiatica* using Illumina paired-end sequencing technology. It displayed a typical quadripartite structure with a sequence length of 158,794 bp, comprised of a large single copy of 88,196 bp, small single-copy of 18,448 bp, and a pair of inverted repeat regions of 26,075 bp each. The cp genome contained 35 tRNA genes, eight rRNA genes, and 86 protein-coding genes with an overall GC content of 36.8%. Phylogenetic analysis revealed a close evolutionary relationship between *B. racemosa* and *B. fusicarpa*, forming a monophyletic group with *B. asiatica* with high bootstrap support, thereby enhancing our understanding of the phylogeny, systematics, and genetics of Asian Lecythidaceae species.

## Introduction

*Barringtonia asiatica* (L.) Kurz (1876), commonly known as the fish poison tree, is an important beach forest species from the Planchonioideae subfamily of Lecythidaceae (Mori et al. 2007). The species is found extensively across wet tropical biomes in coastal regions of tropical Asia and the Pacific (POWO 2024). Native to the Philippines (Pelser et al. 2011), it is locally known as ‘botong’ and is widely cultivated both as a medicinal and ornamental tree. Various parts of the plant have been utilized in traditional medicine, its leaves can be used either fresh to ease rheumatism or heated to relieve stomach discomfort, while the seeds are employed as a vermifuge (Yaplito 2001); the bark is used for tuberculosis and the fruit juice is applied to treat scabies (Quisumbing 1978). The tree produces a large quantity of phytochemicals including alkaloids, flavonoids, and glycosides, which are believed to be responsible for its various pharmacological activities (Umaru et al. 2018). It is also a source of important bioactive compounds that have antibacterial, antifungal, and anti-inflammatory activities (Khan and Omoloso 2002; Umaru et al. 2019; Kong et al. 2020). Moreover, the tree is widely cultivated for its attractive flowers and foliage, and as a shade tree and is given priority for planting along seaside gardens and landscapes in tropical and subtropical regions (Kindt et al. 2021). Despite its long history of traditional medicinal use for treating a wide range of conditions and potential biomedical applications, the characterization of its complete chloroplast genome sequence has not been reported. Currently, only two plastomes from the genus *Barringtonia* have been published (Yu et al. 2017). Hence, in this study, we assembled, annotated, and analyzed the complete chloroplast genome sequence of *B. asiatica* to enhance our understanding of its phylogeny, systematics, and genetics of Asian Lecythidaceae species.

## Materials and methods

### Plant material

The study utilized disease-free leaves of *B. asiatica* (2022-001) collected from a mature tree planted in front of the old Graduate School Office, University of the Philippines Los Baños campus (14° 09′ 50″ N, 121° 14′ 23″ E) with proper permission from the dean. The prepared voucher specimen (ICROPS1449) was deposited at the Philippine Herbarium of Cultivated Plants of the Institute of Crop Science at UPLB (https://cafs.uplb.edu.ph/icrops/, Curator Dr. Renerio P. Gentallan Jr., rpgentallan@up.edu.ph). To validate that the genotype corresponds to a specific reference specimen archived in the herbarium, the morphology of the plant was characterized using delineating characters based on the monograph (Payens 1967) and revision (Prance 2012) of the genus *Barringtonia* J.R.Forst. & G.Forst. (1776). The voucher specimen information: PHILIPPINES. Laguna: Los Baños, 9 July 2022, Timog, Bartolome, and Gentallan 2022-001 (ICROPS1449).

### DNA extraction and sequencing

Total genomic DNA was extracted from 0.5 g of fresh leaves using a modified cetyltrimethylammonium bromide (CTAB) protocol (Doyle and Doyle 1987). The quantity of the sample was verified using A260/280 ratio with DeNovix DS-11+ spectrophotometer and the quality was checked through agarose gel electrophoresis using 1% agarose. Subsequently, an ample and intact DNA sample was submitted to NovogeneAIT Genomics Singapore PTE LTD (Singapore) for complete chloroplast sequencing using the Illumina HiSeq-PE150 platform (Illumina Inc., San Diego, CA). Fastp version 0.20.0 (Chen et al. 2018) was used for read-quality control, resulting in a total of 23,299,266 cleaned reads after successive filtering of the 150-bp paired-end raw reads.

### Chloroplast genome assembly and annotation

Using GetOrganelle v1.7.5 software, we assembled the chloroplast genome of *B. asiatica*, resulting in a circular genome. The specifications for the chloroplast genome assembly followed the suggested assembly recipe in GetOrganelle for the embryophyte plant plastid genome (plastome), using 2G of raw data from 150-bp paired-end reads (Jin et al. 2020). Subsequently, the circularized genome underwent annotation and mapping using CPGAVAS2 (Shi et al. 2019) and was visualized through Chloroplast Genome Viewer (CPGView) (Liu et al. 2023). The assembled chloroplast genome sequence was submitted to GenBank with accession no. ON674119/NC_070213 at the National Center for Biotechnology Information (NCBI).

### Phylogenetic analysis

To determine phylogenetic relationships, complete chloroplast genomes of *B. asiatica* and of three related species from the Lecythidaceae family were used as the in-group, along with *Linanthus parryae* (Polemoniaceae) and five *Impatiens* species (Balsaminaceae) from the same order, Ericales, served as the sister group and outgroup, respectively. The selection of the taxonomic grouping was based on the phylogenetic relationships of Lecythidaceae family established through a cladistic analysis using rbcL sequence and morphological data (Morton et al. 1997). The complete plastome sequences of all nine species were downloaded from NCBI and were aligned using MAFFT v. 7.4 (Katoh and Standley 2013). A phylogenetic tree was constructed using the maximum-likelihood method with the best-fit model of GTR + G (Nei and Kumar 2000) and implemented in MEGA version 11.0.13 (Tamura et al. 2021). The bootstrap analysis was performed with 1000 replicates.

## Results

### Morphological characteristics of the plant material

**Figure 1.**
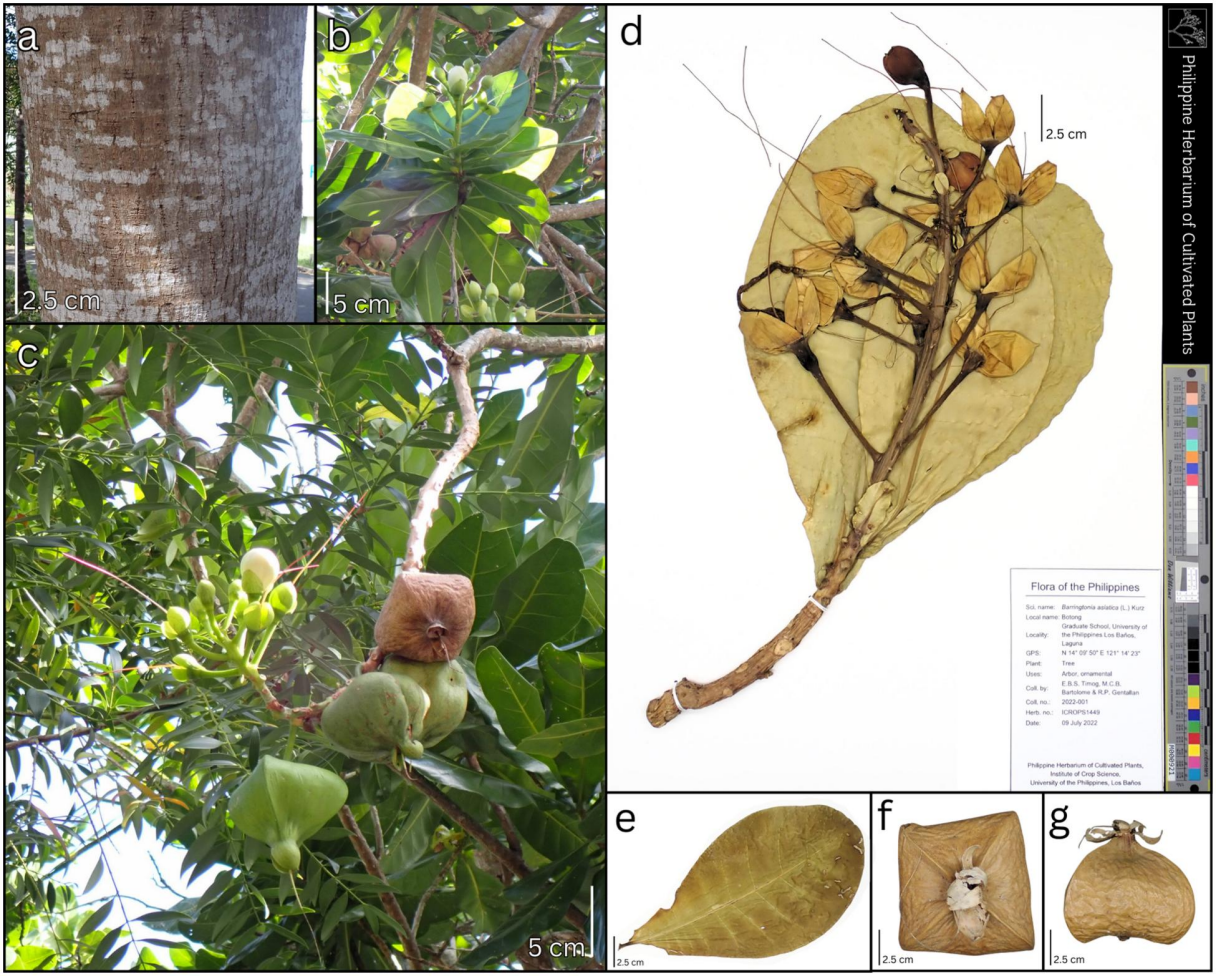
Morphological characteristics and herbarium specimen of *B. asiatica* (ICROPS1449) used for chloroplast genome sequencing. Live (a) bark, (b) leaves, and (c) flowers and fruits; preserved (d) flowering twig, (e) adaxial leaf, and fruit: (f) top view and (g) side view. Photo source: ©CBGR Laboratory, ICropS, CAFS, UPLB.

*B. asiatica* is a large tree, up to 7 m tall with fissured bark (Figure 1(a)) with leaves spirally arranged, sessile to subsessile, petioles 0–0.7 cm long, obovate to obovate-oblong, 20–26 × 10–14 cm, leathery, shiny, dark green above and lighter beneath, glabrous on both sides, base cuneate, margin entire, apex obtuse, or broadly rounded (Figure 1(b,d,e)). The inflorescences are racemes, erect, and mostly terminal, 5–15 cm, 5–10(–20)-flowered; flowers pedicellate (4–8 cm) with undivided calyx; and petals 4, white, ovate or elliptic, 5–6 cm. The fruits are buoyant with a wide pyramidal shape, smooth surface, 9–11 cm, narrowing toward the top and topped with a calyx (Figure 1(c,f,g)). The characteristics of the utilized plant in the plastome assembly and the herbarium specimen ICROPS1449 (Figure 1(d)) were consistent with the range of measurements and descriptions established for *B. asiatica* provided in the monograph and revision of the genus *Barringtonia* (Payens 1967; Prance 2012).

### Chloroplast genome characteristics

**Figure 2.**
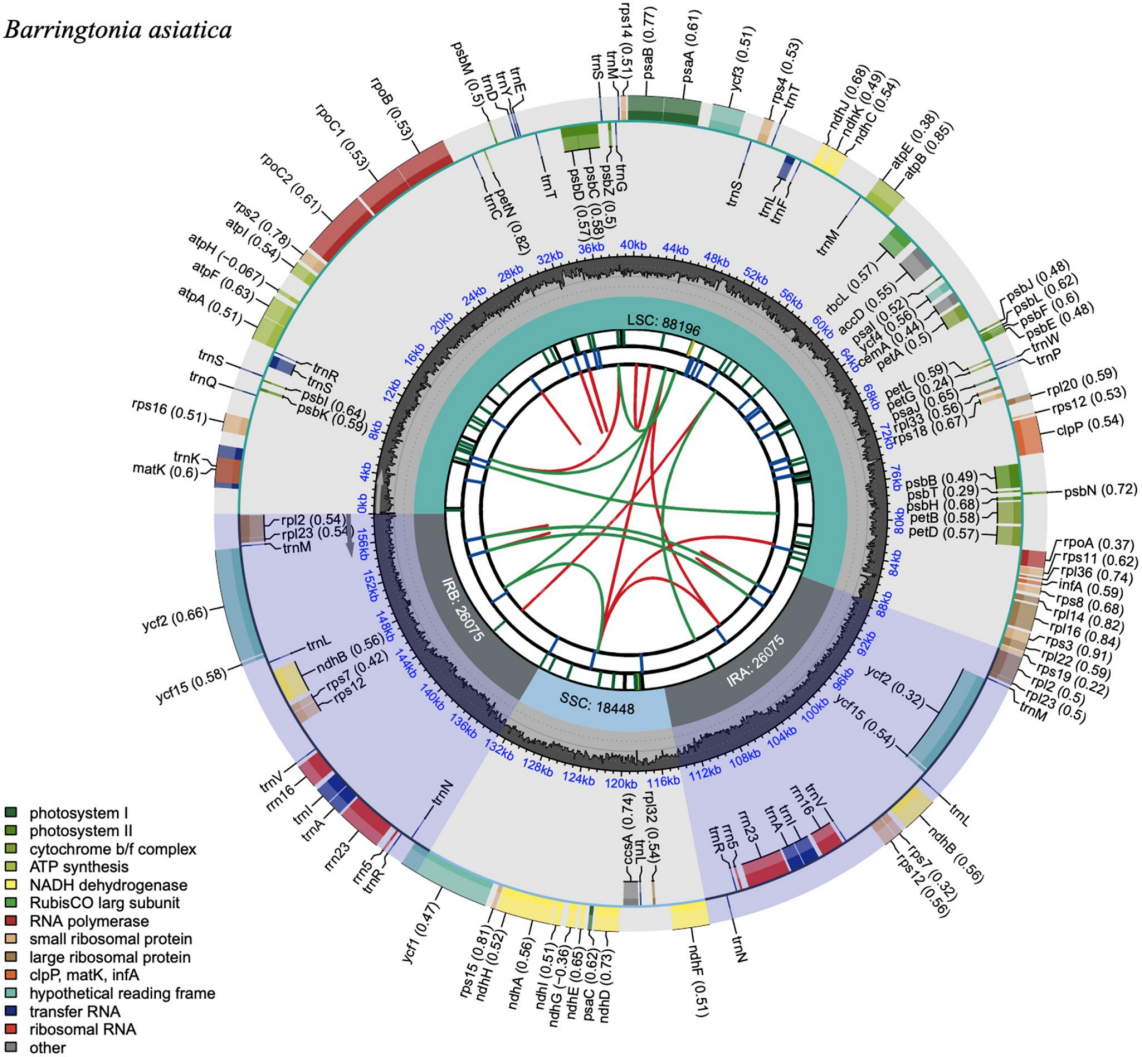
The chloroplast genome (plastome) map of *B. asiatica*. The plastome map is segmented into six concentric circles, each representing different features. Starting from the innermost circle, the first one illustrates dispersed repeats linked by red arcs (forward repeats) and green arcs (palindromic repeats). The second circle depicts the long tandem repeats through short blue bars. The third circle exhibits simple sequence repeats (SSRs) color-coded according to repeat unit size (RUS) (black for complex repeats; green for RUS = 1; yellow for RUS = 2; and blue for RUS = 4). The fourth circle showcases the four regions of the plastome (LSC, SSC, IRa, and IRb) along with their respective sizes. The fifth circle presents the GC content across the genome. The sixth circle displays the genes, their codon usage bias in parentheses, and their color-coded functional group, with a functional group legend provided at the bottom left corner. Genes located in the inner and outer circles are transcribed clockwise and counterclockwise, respectively.

The assembled whole chloroplast genome of *B. asiatica* displayed a typical quadripartite structure (Figure 2; Figure S1). Its complete plastome had a sequence length of 158,794 bp, consisting of a large single-copy (LSC: 88,196 bp), a small single-copy (SSC: 18,448 bp), and two inverted repeat regions (IRa and IRb: 26,075 bp each). The overall GC content of the chloroplast genome was 36.8% with base compositions of 31.3% A, 18.7% C, 18.1% G, and 31.9% T. A total of 127 functional genes were found, comprising 35 tRNA genes, eight rRNA genes, and 86 protein-coding genes. These include six conserved ORFs (*ycf1*, *ycf15* (×2), *ycf2* (×2), and *ycf4*), 27 genes for self-replication, 45 genes for photosynthesis, and six other genes (*accD*, *ccsA*, *cemA*, *clpP*, *infA*, and *matK*). Furthermore, *rps12* is a trans-splicing gene (Figure S2) and a total of 13 cis-splicing genes were identified, of which nine (*rps16*, *atpF*, *rpoC1*, *ycf3*, *clpP*, *petB*, *petD*, *rpl16*, and *ndhA*) were unique and two (*rpl2* and *ndhB*) were duplicates (Figure S3).

### Phylogenetic analysis

**Figure 3.**
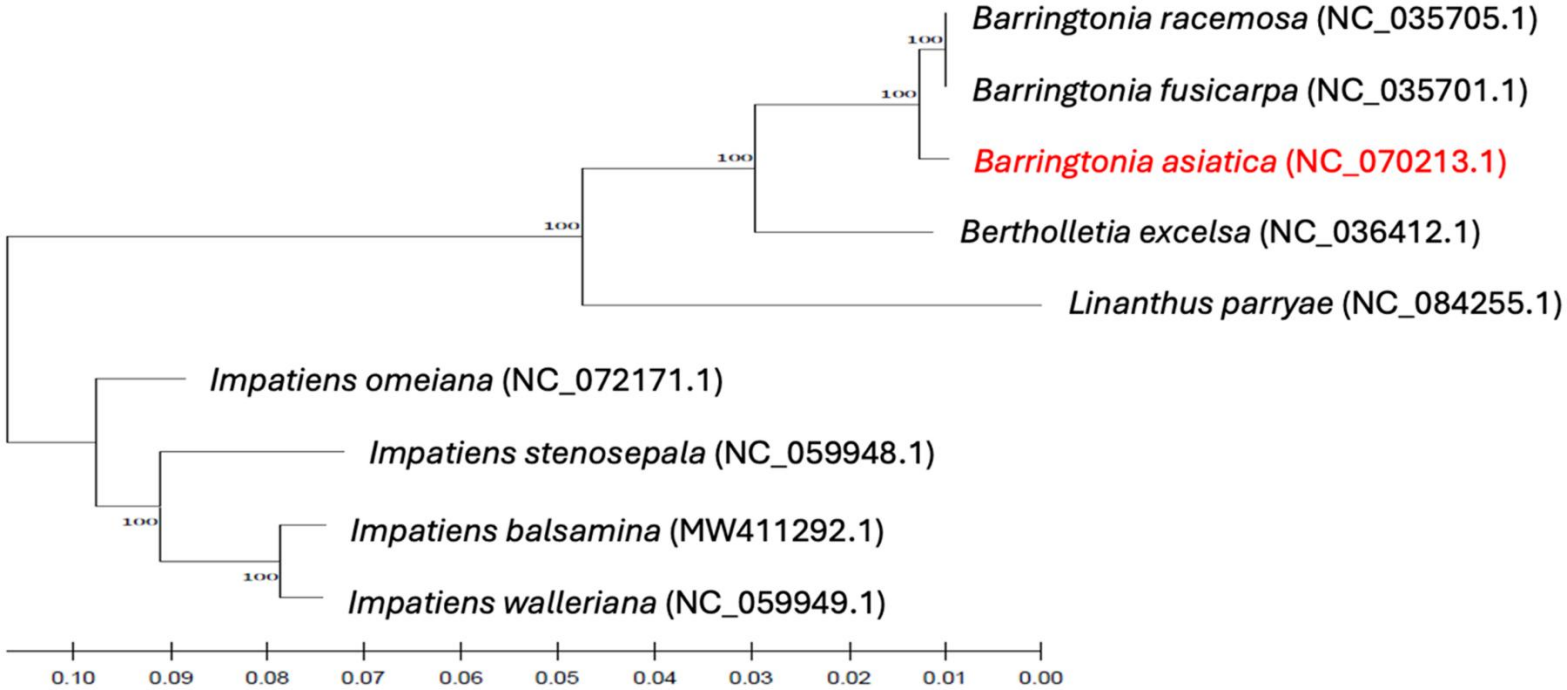
Phylogenetic tree reconstructed using maximum-likelihood (ML) method based on complete chloroplast genome sequences of *B. asiatica* and three other Lecythidaceae species with *Linanthus parryae* (Polemoniaceae) as the sister group and *Impatiens* spp. (Balsaminaceae) as the outgroup taxa. The numbers above the lines represent ML bootstrap values. The following sequences were used: *Barringtonia asiatica* NC_070213.1 (this publication), *Barringtonia fusicarpa* NC_035701.1 (Yu et al. 2017), *Barringtonia racemosa* NC_035705.1 (Yu et al. 2017), *Bertholletia excelsa* NC_036412.1 (unpublished), *Linanthus parryae* NC_084255.1 (unpublished), *Impatiens omeiana* NC_072171.1 (unpublished), *Impatiens stenosepala* NC_059948.1 (Luo et al. 2021), *Impatiens balsamina* MW411292.1 (Luo et al. 2021), and *Impatiens walleriana* NC_059949.1 (Luo et al. 2021). Scale bar refers to evolutionary distance.

The reconstructed phylogram suggested a close evolutionary relationship between *B. racemosa* and *B. fusicarpa*, forming a monophyletic group with *B. asiatica* supported by a high bootstrap value of 100% (Figure 3). The phylogenetic results of this study strengthen those previously reported by Mori et al. (2007) using combined DNA barcodes regarding the evolutionary relationships among species under the Lecythidaceae subfamily Planchonioideae (formerly Barringtonioideae).

## Discussion and conclusions

*Barringtonia asiatica* is a beach forest plant native to the Philippines, known for its medicinal and ornamental uses. It has been utilized in traditional folk medicine and has great potential as a source of phytochemicals and bioactive compounds for various biomedical applications. However, its genomic resources are still unavailable. This is the first detailed characterization of the complete chloroplast genome of *B. asiatica*. The sequenced, *de novo* assembled, and annotated plastome has a sequence length of 158,794 bp, which is shorter than the chloroplast genomes of *B. racemosa* (159,002 bp) and *B. fusicarpa* (158,927 bp) available in NCBI (Yu et al. 2017). The chloroplast genome of *B. asiatica* exhibits a generally conserved plastome structure, like that found across other species. On the other hand, the phylogenetic analysis using the available whole chloroplast genomes supported the previously established monophyly of the Lecythidaceae subfamily Planchonioideae (formerly Barringtonioideae), as determined by combined DNA barcodes. This study presents a new plastome sequence for the genus *Barringtonia* that will contribute to the elucidation of the evolutionary relationship among taxa within the Lecythidaceae family of the order Ericales and serve as a resource for future genetic studies.

## Supplementary Material

Supplementary Materials.docx

## Data Availability

The genome sequence data supporting the findings of this study are openly available in GenBank of NCBI at https://www.ncbi.nlm.nih.gov/nuccore/NC_070213.1/ under the accession no. ON674119/NC_070213.1. The associated BioProject, SRA, and BioSample numbers are PRJNA1096474, SRX24164366, and SAMN40757201, respectively.
