# Supplementary material for: The complete chloroplast genome of Barringtonia asiatica (L.) Kurz (Lecythidaceae)
Source: Mitochondrial DNA B Resour. 2025 Jan 30;10(2):149–53. doi: 10.1080/23802359.2025.2457456 (PMC11784068; doi:10.1080/23802359.2025.2457456)
Supplement: Supplementary Materials.docx [file TMDN_A_2457456_SM0560.docx]

**Supplementary Materials**

**
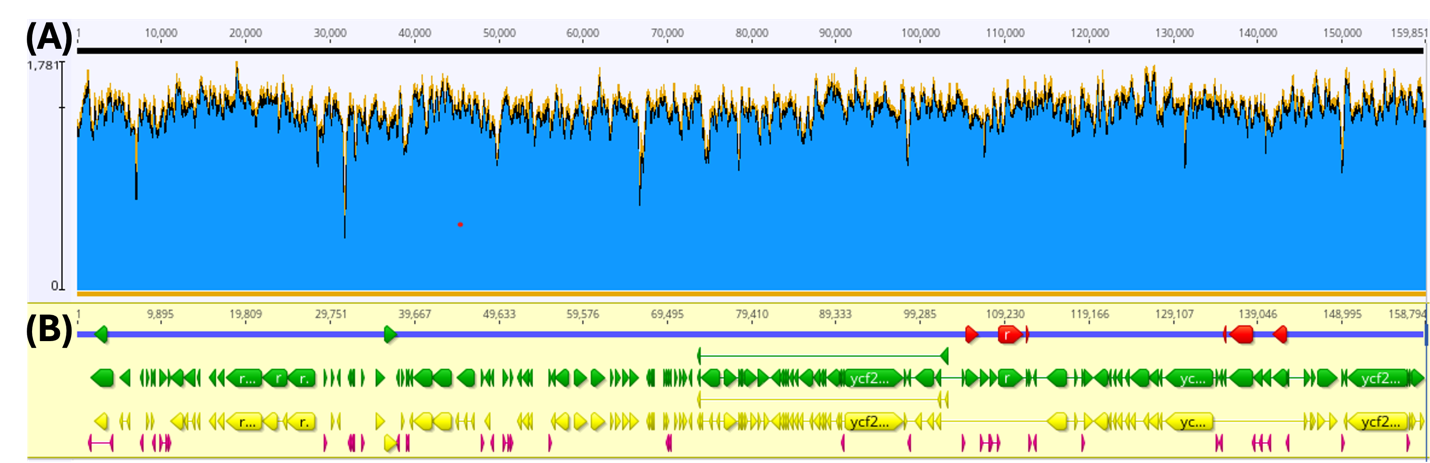
**

**Figure S1.** Depth of coverage plot (A) and physical map of cpDNA (B). The reads from Illumina NGS sequencing were remapped on the cp genome sequence of *Barringtonia asiatica* (NC_070213.1) using Bowtie2 as implemented in Geneious prime 2024.


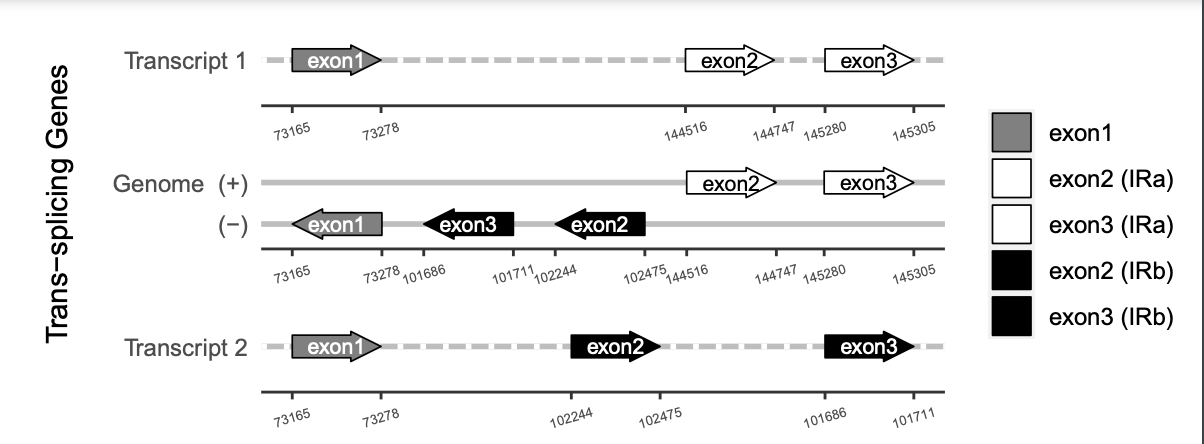


**Figure S2.**  Structure of trans-splicing genes in the chloroplast genome of *B. asiatica*, generated using GeSeq annotation and CPGView, reveals the presence of three unique exons, two of which are duplicated in the inverted repeat (IR) regions. (Note: this trans-spliced region was not annotated in CPGAVAS2).


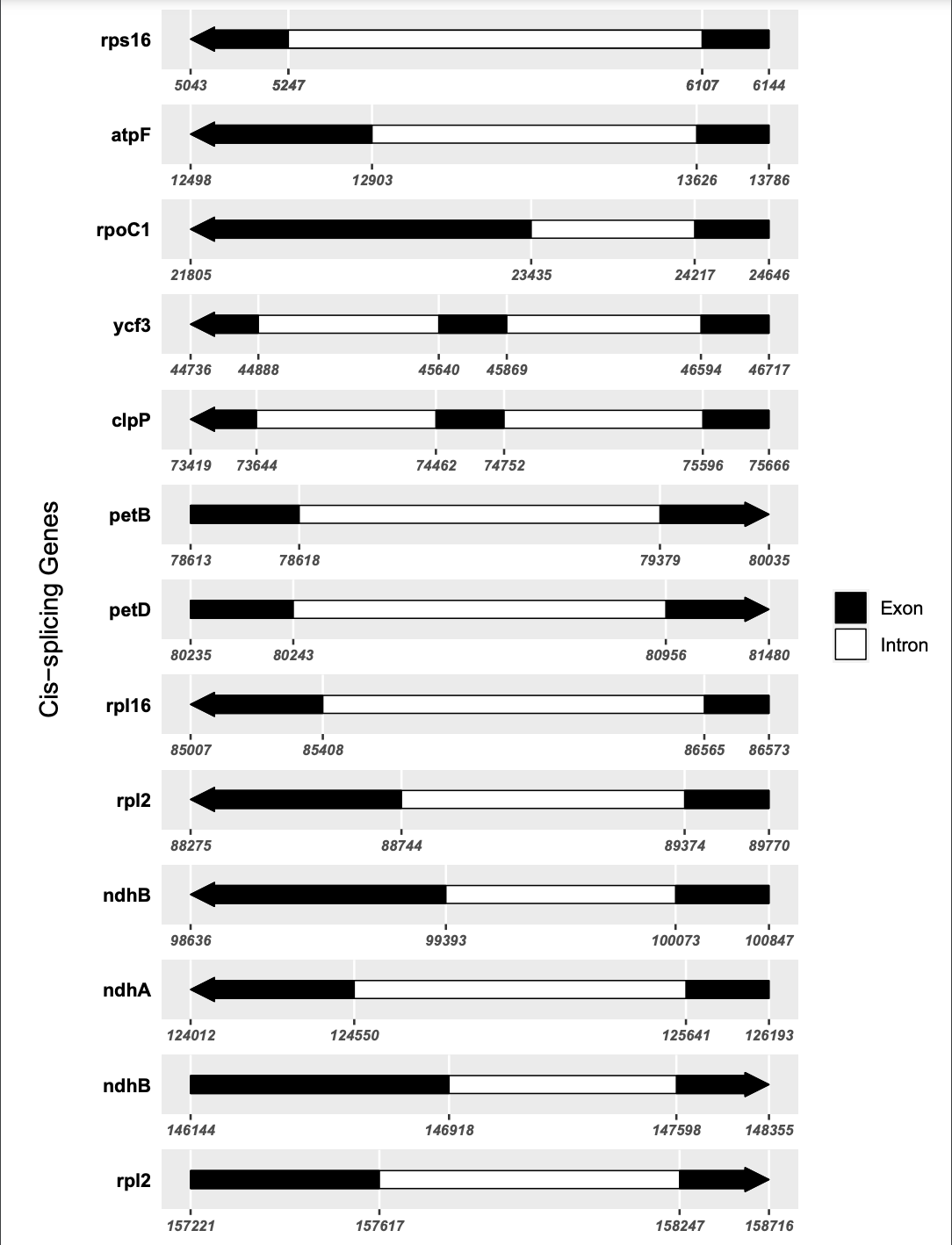


**Figure S3.** A schematic illustration of the cis-splicing genes (CDS) of the chloroplast genome of *B. asiatica* using CPGView which depicts the gene arrangement mirroring the order found in the chloroplast genome. Gene names appear on the left side, while their corresponding structures are displayed on the right. Directionality is indicated by arrows. Exons and introns are depicted in black and white, respectively, though their lengths are not drawn to scale.
